# Antifungal Effect of Penicillamine Due to the Selective Targeting of L-Homoserine *O*-Acetyltransferase

**DOI:** 10.3390/ijms23147763

**Published:** 2022-07-14

**Authors:** Aleksandra Kuplińska, Kamila Rząd, Marek Wojciechowski, Sławomir Milewski, Iwona Gabriel

**Affiliations:** Department of Pharmaceutical Technology and Biochemistry, Gdansk University of Technology, 80-233 Gdansk, Poland; aleksandra.kuplinska@pg.edu.pl (A.K.); kamila.rzad@pg.edu.pl (K.R.); marek.wojciechowski@pg.edu.pl (M.W.); slawomir.milewski@pg.edu.pl (S.M.)

**Keywords:** L-homoserine *O*-acetyltransferase, *Candida albicans*, antifungal target, L-methionine biosynthesis

## Abstract

Due to the apparent similarity of fungal and mammalian metabolic pathways, the number of established antifungal targets is low, and the identification of novel ones is highly desirable. The results of our studies, presented in this work, indicate that the fungal biosynthetic pathway of L-methionine, an amino acid essential for humans, seems to be an attractive perspective. The *MET2* gene from *Candida albicans* encoding L-homoserine *O*-acetyltransferase (*Ca*Met2p), an enzyme catalyzing the first step in that pathway, was cloned and expressed as the native or the oligo-His-tagged fusion protein in *Escherichia coli*. The recombinant enzymes were purified and characterized for their basic molecular properties and substrate specificities. The purified *MET2* gene product revealed the appropriate activity, catalyzed the conversion of L-homoserine (L-Hom) to *O*-acetyl-L-homoserine (OALH), and exhibited differential sensitivity to several L-Hom or OALH analogues, including penicillamine. Surprisingly, both penicillamine enantiomers (L- and D-Pen) displayed comparable inhibitory effects. The results of the docking of L- and D-Pen to the model of *Ca*Met2p confirmed that both enantiomeric forms of the inhibitor are able to bind to the catalytic site of the enzyme with similar affinities and a similar binding mode. The sensitivity of some fungal cells to L-Pen, depending on the presence or absence of L-Met in the medium, clearly indicate Met2p targeting. Moreover, *C. glabrata* clinical strains that are resistant to fluconazole displayed a similar susceptibility to L-Pen as the wild-type strains. Our results prove the potential usefulness of Met2p as a molecular target for antifungal chemotherapy.

## 1. Introduction

Mycoses are caused by various fungal species; however, life-threatening systemic fungal diseases are mainly entailed by human pathogenic yeasts belonging to the genera *Candida*, especially *C. albicans* and *C. glabrata*, the filamentous fungi of *Aspergillus* spp., and *Cryptococcus* spp. [1]. *C. albicans* is considered as the fourth most popular etiological agent of nosocomial infections worldwide [2]. In recent years, a rising number of invasive fungal infections in immunocompromised patients has been observed, which is also correlated with increased mortality rates [3]. Moreover, the uncontrolled use of some antifungals has become one of the factors stimulating the selection of resistant fungal cells [4]. The current methods of treatment of systemic mycoses remain unsatisfactory, mainly due to the very limited repertoire of effective antifungal chemotherapeutics. The so-called “golden standard”, polyene macrolide antibiotic amphotericin B, the most effective antifungal agent used in clinical practice, exhibits severe mammalian toxicity, especially nephrotoxicity. On the other hand, synthetic “azole” antifungals, including fluconazole, as well as semisynthetic echinocandins, especially caspofungin or 5-flucytosine, display limited effectiveness, mainly due to emerging fungal resistance. None of the drugs used in the clinical treatments of disseminated mycoses fulfil all of the features of the “ideal” antifungal. Such a drug should demonstrate a fungicidal mode of action against the broadest possible spectrum of fungal pathogens, should display the lowest possible toxicity towards the mammalian host, and should exhibit the lowest ability to induce fungal resistance. For the above reasons, there is a growing need for novel and more specific antifungal drugs, especially these that are aimed at new molecular targets. Unfortunately, due to the high similarity of fungal and mammalian cells, the number of identified antifungal targets is still very low. 

Only very few of 130 *C. albicans* genes identified as essential and not having counterparts in mammalian systems [5] have been proposed so far as possible targets for novel antifungals. From this point of view, enzymes catalyzing particular unique steps of fungal biosynthetic pathways of nine amino acids essential for humans seem to be an attractive perspective [6,7]. The development of an antifungal agent that acts as an inhibitor of the fungal enzyme involved in the biosynthesis of any human-essential amino acid might result in its selective toxicity. The fungi-specific pathways of L-methionine and L-tryptophan biosynthesis are considered as the most promising sources of potential targets, since the serum levels of these two human-essential amino acids are especially low [8], well below the concentrations needed to rescue Met or Trp auxotrophy.

**Figure 1:**
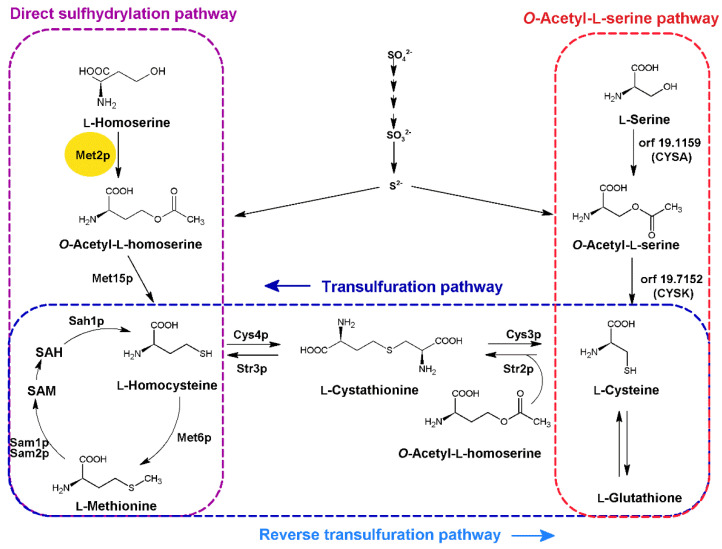
Fungal pathways of L-methionine and L-cysteine biosynthesis for incorporation of inorganic sulfur. Enzymes marked in bold: Met2p EC 2.3.1.31 homoserine *O*-acetyltransferase; Met15p EC 2.5.1.49, EC 2.5.1.47 bifunctional *O*-acetyl-L-homoserine/*O*-acetyl-L-serine sulfhydrylase; Met6p EC 2.1.1.13 methionine synthase; Sam1p, Sam2p EC 2.5.1.6 methionine adenosyl transferase; Sah1p EC 3.3.1.1 adenosyl homocysteinase; Cys4p EC 4.2.1.22 cystathionine-β-synthase; Str3p EC 4.4.1.8 cystathionine-β-lyase; Cys3p EC 4.4.1.1 cystathionine-γ-lyase; Str2p EC 2.5.1.48 cystathionine-γ-synthase; orf 19.1159 putative homologue of the *A. nidulans* L-serine *O*-transacetylase (CYSA); orf 19.7152 putative homolog of *Aspergillus O*-acetyl-L-serine sulfhydrylase (CYSK).

L-methionine is synthesized in fungal cells through the direct sulfhydrylation pathway or the transsulfurylation pathway, from L-homoserine or L-cysteine, respectively (Figure 1). The first step of the direct sulfhydrylation pathway is catalyzed by L-homoserine *O*-acetyltransferase EC 2.3.1.31 (Met2p), converting L-homoserine (L-Hom) to *O*-acetyl-L-homoserine (OALH).

Met2p is undoubtedly a potential molecular target for novel antifungal chemotherapeutics. Nazi et al. discovered that this enzyme is essential for the virulence of *Cryptococcus neoformans* in a mouse inhalation model [9]. The disruption of the *MET2* gene in this fungus caused methionine auxotrophy that could be rescued via the addition of >60 µM L-methionine to the growth medium [9]. This concentration level is at least two-fold higher than the normal concentration of that amino acid in human serum [8]. Additionally, other studies revealed that *Candida albicans*, *Candida guilliermondii*, and *Saccharomyces cerevisiae* mutant cells depleted in *MET2* genes turned out to be methionine auxotrophs [10,11,12]. L-homoserine *O*-acetyltransferase has therefore been proposed as one of the potential molecular targets for antifungal drugs [13]. Nevertheless, the identified heterocyclic inhibitor of Met2p demonstrated very poor, if any, growth inhibitory activity against *C. neoformans* [9], possibly due to poor bioavailability.

It is worth mentioning that L-homoserine *O*-acetyltransferase is also essential in bacterial cells. *Mycobacterium tuberculosis* cells lacking the *META* gene, encoding the bacterial homolog of Met2p, were unable to induce infection in an vivo mouse inhalation model or proliferate inside human macrophages, and they could not grow in L-methionine-free medium [14]. Chaton et al. suggested that the active site of mycobacterial Met2p is highly druggable [15].

In this paper, we present the results of our studies on the determination of the potential usefulness of L-homoserine *O*-acetyltransferase as a molecular target in antifungal chemotherapy. The *MET2* gene from *C. albicans* was cloned and expressed as the native or the oligo-His-tagged fusion protein in *E. coli*. The recombinant enzymes were purified and characterized for their basic molecular properties and substrate specificities, and the enzyme inhibitory potentials of several L-homoserine or L-methionine structural analogs were evaluated.

## 2. Results and Discussion

### 2.1. Identification of CaMET2 Gene and Bioinformatical Analysis of the Predicted Gene Product

The *MET2* gene (orf19.2618; CR_02170W_A, CR_02170W_B), encoding putative L-homoserine *O*-acetyltransferase from *C. albicans* (*Ca*Met2p), was retrieved from the Candida Genome Database [16]. The expected gene product, Met2p, is a 409 amino acid polypeptide with a pI value of 5.20 and a theoretical molecular weight of 45.418 kDa (ProtParam analysis) [17]. The BLAST analysis and a multiple sequence alignment revealed homology (percentage of identity shown in brackets) between the *Ca*Met2p amino acid sequence and the sequences encoding L-homoserine *O*-acetyltransferases; with defined crystal structures from *Haemophilus influenzae* (34%) (PDBID: 2B61), *Staphylococcus aureus* (28%) (PDBID: 4QLO), *Mycobacterium smegmatis* (32%) (PDBID: 6IOG), and putative Met2p from *Saccharomyces cerevisiae* (53%) (Saccharomyces Genome Database: YNL277W); and the presence of conserved and important residues.

**Figure 2:**
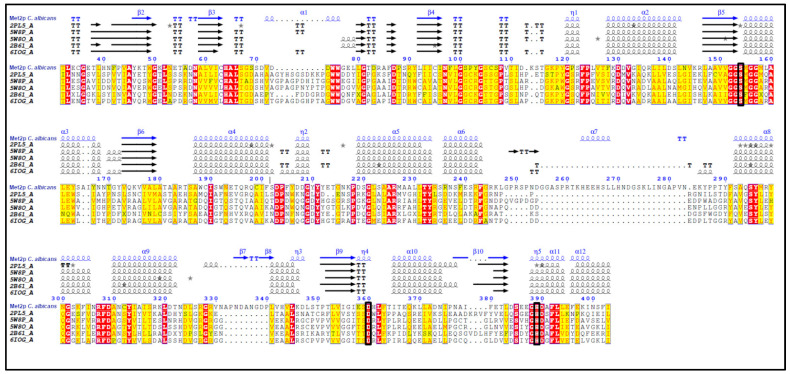
The multiple sequence alignment of the amino acid sequence of *Ca*Met2p and homologous sequences of L-homoserine *O*-acetyltransferases, with determined 3D structures from the PDB database. Comparison of the predicted secondary structure of *Ca*Met2p with those of the other known L-homoserine *O*-acetyltransferases. Analysis performed using the ENDscript program [19]. Sequence numbering refers to *Ca*Met2p. Residues 1–31 and 404–409 are not present. Colors: red—identity, yellow—similarity, symbols: α, α-helix; β, β-sheets; η, 3_10_-helices; TT, strict β-turns; ★, residues with alternate conformations. The probable catalytic amino acids: Ser^154^, Asp^360^, and His^389^ in the *Ca*Met2p sequence are shown in the black square frame. PDB codes: 5W8O *Mycobacterium hassiacum*, 5W8P *Mycobacterium abscessus*, 6IOG *Mycobacterium smegmatis*, 2B61 *Haemophilus influenza*, and 2PL5 *Leptospira interrogans*.

The Standard Protein BLAST analysis of amino acid sequences of proteins homologous to *Ca*Met2p (≥50.34% identity) from 59 microorganisms enabled us to perform a multiple sequence alignment (result presented in Figure S1) and prepare a phylogenetic tree (Figure S2). Several highly conserved sequences were identified, among which the Gly-Gly-Ser-Met-Gly-Gly-Met motif was distinguished. This is a characteristic structural element termed the “nucleophilic elbow”, specific for the α/β-hydrolase fold superfamily of enzymes, which contains one nucleophilic residue from the Ser-His-Asp catalytic triad [18], particularly Ser^154^ in the *Ca*Met2p sequence. This residue corresponds to Ser^151^, Ser^143^, and Ser^153^, from *Mycobacterium smegmatis* (PDBID: 6IOG), *Haemophilus influenza* (PDBID: 2B61), and *Leptospira interrogans* (PDBID: 2PL5), respectively (Figure 2). 

The results from these two alignments allowed for the identification of two other residues in the *Ca*Met2p sequence constituting the catalytic triad, namely Asp^360^ and His^389^. Asp^360^ is one of the residues of the conserved motif Ser- Asp-X-Leu-Phe, and His^389^ is included in the sequence Glu/Asp-Gly-His-Asp-Ala/Gly-Phe-Leu-Leu/Ile. Asp^360^ and His^389^ correspond to Asp^314^/His^344^, Asp^304^/His^337^, and Asp^311^/His^344^ from the catalytic triads of *Mycobacterium smegmatis, Haemophilus influenzae*, and *Leptospira interrogans* Met2p, respectively. A comparison of the predicted secondary structure of *Ca*Met2p with the known crystal protein structures from the PDB database is shown in Figure 2. The sequence regions predicted as α-helices and β-sheets in *Ca*Met2p are 61–73.5% and 51–60%, respectively, aligned with their counterparts in the sequences of homologous proteins.

**Figure 3:**
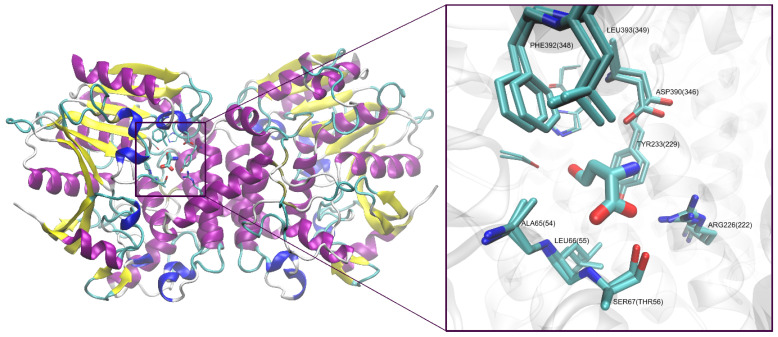
Structure of *C. albicans* L-homoserine *O*-acetyltransferase dimer based on the AlphaFold model and the template structure of the enzyme from *M. smegmatis* (PDBID: 6IOH). To visualize similarity of both binding sites and to obtain the proper ligand orientation for reference and docking results validation, both structures were superimposed with natural substrate of the enzyme (L-HOM), found in the 6IOH pdb file, with neighboring binding site residues are drawn as sticks. Residues are labeled according to the *C. albicans* sequence numbering. Values in parentheses resemble the numbering of *M. smegmatis* enzyme. Catalytic triad comprising of Ser^154^(Ser^151^), His^389^(His^344^), and Asp^360^(Asp^314^) is visible in the background, and is depicted with the thinnest sticks, without labels, to keep picture clarity.

Since there is no available X-ray structure of *C. albicans* L-homoserine *O*-acetyltransferase, the structure of the enzyme from *M. smegmatis* (PDBID: 6IOH) was used as a template to prepare the AlphaFold model of the *C. albicans* enzyme [20]. A superposition of binding sites of L-homoserine *O*-acetyltransferase from *C. albicans* and *M. smegmatis* showed that despite the barely 32% identity of sequences, the binding sites of these two enzymes are very well conserved (Figure 3).

Six out of 10 residues, namely Thr^50^, Asp^52^, Arg^61^, Ser^143^, Arg^212^, Tyr^219^, Tyr^294^, Asp^304^, His^337^, and Asp^338^, forming the intramolecular tunnel via the juxtaposition of the two domains of the *H. influenzae* Met2p [21], are conserved in *Ca*Met2p (Ser^154^, Arg^225^, Tyr^233^, Asp^360^, His^389^, and Asp^390^) (Figure 2), as well as in Met2p from the microorganisms of the *Ascomycetes* group (Figure S1). Notable differences concern Ser^67^ and Ser^69^ in the *Ca*Met2p sequence, corresponding to Thr^50^ and Asp^52^ in *Hf*Met2p, though Ser^69^ is well conserved in *Ascomycetes* Met2p sequences (Figure S1).

The most conserved motifs of protein amino acid sequences from *Ascomycota* (Figure S1) group were analyzed with the use of previously published methods [22,23,24], and were selected for comparison to the sequences of enzymes with resolved crystal structures from chosen organisms (Figure S3). The block diagram shows the location of the characteristic motifs in the sequences of L-homoserine *O*-acetyltransferases from the analyzed organisms. The *Ca*Met2p sequence represents the *Ascomycota* group. Those motifs included residues of the catalytic triad (Figure S3).

In those motifs, it is possible to observe a single difference in a residue of the catalytic side, which is formed by the amino acid residues stabilizing an intermediate product of the enzymatic reaction. In *H. influenzae*, these residues are Leu^49^, Phe^144^, and Arg^212^, and in Met2p from *C. albicans*, there are Leu^66^, Met^155^ (instead of Phe; in the first GGSM/F motif), and Arg^225^. All of these residues (Leu, Met, and Arg) are conserved in the *Ascomycetes* group (Figure S1).

### 2.2. Subcloning, Protein Expression, and Purification

The *CaMET2*, *CaMET2NH*, and *CaMET2CH* genes, encoding, respectfully, *C. albicans* wild-type L-homoserine *O*-acetyltransferase (*Ca*Met2p), N-terminus His-tagged L-homoserine *O*-acetyltransferase (*Ca*Met2NHp), and C-terminus His-tagged L-homoserine *O*-acetyltransferase (*Ca*Met2CHp), were cloned into the pET101/D-TOPO expression vector, yielding the pET101/D-TOPO + *MET2*, pET101/D-TOPO + *MET2NH*, and pET101/D-TOPO + *MET2CH* plasmids. Plasmids containing the *CaMET2* gene with an oligo-Histidine tag added to either the C- or N-terminus, were constructed to facilitate further purification of the protein. Additionally, a mutagenesis procedure was conducted because the *CaMET2* gene contains a single CTG codon at position 490–492, which is translated as L-serine in *C. albicans*, but as L-leucine by *E. coli* cells. The obtained plasmids were sequenced and then *E. coli* BL21 Star (*DE3*) and *E. coli* Rosetta (*DE3*) *pLysS* competent cells were transformed with pET101/D-TOPO + *MET2* and pET101/D-TOPO + *MET2NH*, respectively. The transformed cells were used for the overexpression of the cloned genes in a IPTG-inducible Tabor-Studier system, which allowed for the overproduction of *Ca*Met2p, *Ca*Met2CHp, and *Ca*Met2NHp. Proteins were purified using the AKTA Fast protein liquid chromatography (FPLC) system. HisTrap™ Fast Flow metal ion affinity chromatography was used for *Ca*Met2NHp purification. The target protein was eluted with 250 mM imidazole, with an 86% purity rate. CaMet2p was purified on an ion exchange Resource™ Q column and eluted with 16% NaCl, with a 44% purity rate (Figure S4).

Both proteins submitted to SDS-PAGE electrophoresis resulted in one band of ~45 kDa (Figure S5a). Before the protein purification, the *Ca*Met2NHp production yield and the tightness of the expression system were checked using Western blot analysis (Figure S5b). 

### 2.3. Characterization of CaMet2p Properties

**Figure 4:**
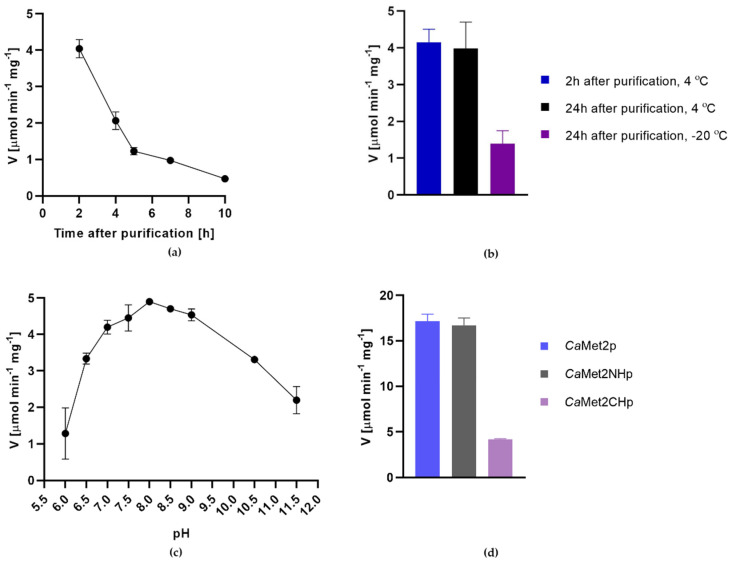
Determination of the optimum conditions for *Ca*Met2p/*Ca*Met2NHp activity: (**a**) Loss of *Ca*Met2NHp activity in time when not supplemented with glycerol; (**b**) effect of glycerol addition as well as storage temperature on *Ca*Met2NHp stability; (**c**) dependence of *Ca*Met2NHp activity on pH; (**d**) comparison of *Ca*Met2p, *Ca*Met2NHp, and *Ca*Met2CHp activities measured at optimal conditions. Data are presented as means of at least three independent experiments (V [µmol·min^−1^·mg^−1^] ± SD).

Three purified enzyme versions, *Ca*Met2p, *Ca*Met2NHp, and *Ca*Met2CHp catalyzed the reaction that utilizes L-Hom and acetyl-coenzyme A (AcCoA) as substrates to produce OALH. Specific activities of the wild-type and N-terminus His-tagged enzyme were at a similar level, whereas they were significantly lower in the case of the C-terminus His-tagged enzyme version (Figure 4d). A low activity of *Ca*Met2CHp enzyme might be due to the presence of the catalytic His^389^ residue that is close to the C-terminus; therefore, the oligo-His tag could disturb substrate access to the active center. The freshly purified *Ca*Met2NHp turned out to be highly unstable, as it had lost its enzymatic activity almost completely by 4 h after purification (Figure 4a). The activity of the wild-type *C*aMet2p activity also decreased, but not so drastically (~30% within 24 h). The stability of *Ca*Met2NHp and *Ca*Met2p could be improved by adding glycerol up to 20% *v/v*. The enzyme preparation remained active for a longer period of time when kept at 4 °C (Figure 4b). A similar loss of activity was observed in the case of the *Ca*Met2p analogs from *S. cerevisiae, Bacillus polymyxa*, and *Brevibacterium flavum*, reported to require the presence of a polyhydroxy agent such as glycerol or sucrose, to ensure a longer stability [25,26,27]. Both *Ca*Met2p and *Ca*Met2NHp showed the highest activity at pH 8.0. An activity assay was conducted at 37 °C (Figure 4c). Consistent with our results, the previous studies characterizing Met2p from *S. cerevisiae* and *S. pombe* also discovered that those enzymatic proteins were most active at pH 7.5–8.0 [18,27].

The kinetic parameters of Met2p from *C. albicans* determined for purified *Ca*Met2p and *Ca*Met2NHp versions are presented in Table 1. The respective parameters determined for both versions are similar, and in the case of the k_cat_ values, almost identical. This indicates that catalytic properties of Met2p are not affected by the presence of the N-terminal oligoHis tag. On the other hand, the K_M_ values appeared to be quite ambiguous compared to the ones determined previously by other authors for *S. cerevisiae* and *S. pombe* Met2p. They equaled 1.00 mM and 1.09 mM for L-Hom, and 0.0270 mM and 0.0209 mM for AcCoA, respectively. Especially the latter are very different from those determined by us for *Ca*Met2p. However, the *S. pombe* Met2p k_cat_ values were equal to 9.6 s^−1^ and 9.3 s^−1^ for L-Hom and AcCoA, respectively, and they were comparable to the k_cat_ of the *C. albicans*-derived enzyme. Although *C. albicans, S. cerevisiae*, and *S. pombe* belong to the same family, the parameters of the same enzyme can still differ between species. Interestingly, the K_M_ values established for Met2p from *Mycobacterium smegmatis* that are equal to 0.06 mM for L-Hom and 0.158 mM for AcCoA [28] are closer to the values obtained by us for *Ca*Met2p.

### 2.4. Penicillamine Inhibits CaMet2p

**Figure 5:**
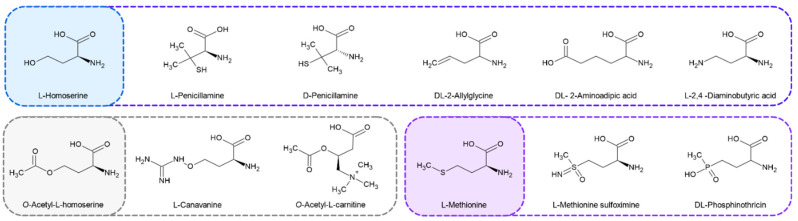
Structures of *Ca*Met2p substrate (L-homoserine), product (*O*-Acetyl-L-homoserine), and the end-pathway product (L-methionine), as well as their analogs as potential inhibitors tested in this study.

Nine compounds, the structures of which are shown in Figure 5, considered as structural analogs of Hom or L-Met, were tested as potential inhibitors of *Ca*Met2NHp. Additionally, we tested whether this enzyme was inhibited by L-Met, i.e., the end-product of the methionine biosynthetic pathway. Only a 17% inhibition of enzyme activity was found for 25 mM L-Met. Undoubtedly, this effect is not physiologically relevant. Therefore, the regulation of enzyme activity through feedback inhibition is unlikely. Similarly, *S. cerevisiae* and *Neurospora crassa* Met2p enzyme showed no evidence of feedback inhibition by L-Met, in contrast to the bacterial *B. polymyxa, B. subtillis*, and *B. flavum* enzymes [27].

**Figure 6:**
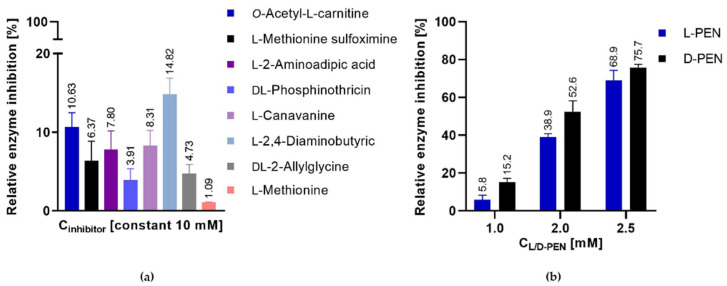
Relative *Ca*Met2NHp inhibition by: (**a**) commercially available Hom analogs and L-Met, 10 mM; (**b**) by L-Pen or D-Pen at 1.0, 2.0, or 2.5 mM concentrations. Data shown are the means of at least three independent experiments ± SD.

Seven out of the nine analogs tested appeared to be poor inhibitors, since at 10 mM, they inhibited the enzyme at 4–15% only (Figure 6a). On the other hand, L-penicillamine (L-Pen) and D-penicillamine (D-Pen) displayed a more pronounced inhibitory effect. D-Pen inhibited the enzyme better than L-Pen at lower concentrations of 1.0 mM and 2.0 mM, with ~15% and ~50%, respectively. However, at 2.5 mM, both L-Pen and D-Pen inhibited *Ca*Met2p at a similar level of ~70% (Figure 6b). The IC_50_ parameter of both compounds, although not determined precisely, could be estimated as ~2.0 mM.

Comparable enzyme inhibitory properties of both penicillamine enantiomers (L- and D-Pen) are quite surprising, in the light of the expected stereospecificity of Met2p. Thus, we decided to perform the molecular docking analysis to check whether both isomers could bind to the active center. Since there is no X-ray structure of *Ca*Met2p available, the structures of the enzyme from *M. smegmatis* (PDBID: 6IOH), as well as the structure of the AlphaFold model (Figure 3) of *Ca*Met2p were used as the receptors for docking. The apparent identity of the active centers of both these matrixes resulted in a very similar outcome for the docking calculations. A closer examination of the L-homoserine binding part of the *Ca*Met2p active site (Figure 3) showed that the residues responsible for anchoring the substrate (namely Arg^226^, Asp^390^, and Ser^67^) are located near the middle of the binding pocket, while the catalytic triad is located deep inside, at the bottom of the binding cavity. The part of the L-homoserine binding site, between these two regions, is formed by the Leu^66^, Ala^65^, Phe^392^, Tyr^233^, and Leu^393^ residues with their sidechains shaping a hydrophobic ring around this area (Figure 3).

**Figure 7:**
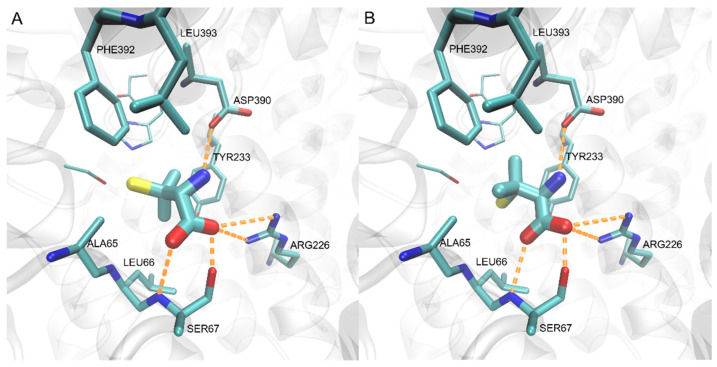
Results of docking for L- (**A**) and D-penicillamine (**B**) to the active site of the modeled *Ca*Met2p. The bound ligands are depicted with thick sticks, and the neighboring active site residues with regular sticks. Catalytic triad comprising of Ser^154^, His^389^, and Asp^360^ sidechains is visible in the background of the binding site, and is drawn with the thinnest sticks. Hydrogen bonds are represented by orange dashed lines.

The results of the docking of L- and D-Pen (Figure 7) to the model of *Ca*Met2p confirmed that both enantiomers are able to bind to the binding site of the enzyme with similar affinities and similar binding modes. There is enough room in the active site to accommodate the changed pose of the ligand, resulting from its different Cα configuration, while the carboxy and alpha-amino moieties are still able to recreate the network of interactions of the natural substrate with the Asp^390^, Arg^226^, and Ser^67^ residues. The binding of the remaining, nonpolar part of the ligands is dominated by nonspecific van der Waals interactions with the surrounding hydrophobic ring, resulting in similar affinities of L- and D-penicillamine.

Penicillamine is a sulfur-containing, non-proteinogenic amino acid that was reported to inhibit several pyridoxal-5′-phosphate (PLP)-dependent enzymes [29]. Both L-Pen and D-Pen can interact with the vitamin cofactor PLP, but L-Pen is generally a more effective inhibitor of PLP-dependent enzymes, because it better mimics the L-amino acid substrates of these enzymes. The inhibitory potential of L-Pen and D-Pen towards Met2p is the first example of PLP-independent enzyme inhibition by these compounds. Our results are particularly interesting in the light of the low cytotoxicity of D-Pen, which is FDA-approved and clinically used as a first-line therapy for patients with Wilson’s disease (WD), which can cause hepatic and neurologic damage due to copper metabolism disorders. D-Pen is used to treat WD because of its capacity to chelate metals [30]. On the other hand, L-Pen is not used clinically, due to its strong inhibition of pyridoxine-dependent enzymes, leading to neurotoxicity in animal experiments [31].

### 2.5. Penicillamine Exhibits Antifungal In Vitro Activity

The antifungal in vitro activities of the Met2p inhibitors, L- and D-Pen, against several *Candida* species and *S. cerevisiae*, were determined by the serial two-fold dilution method in 96-well microtiter plate format. Minimal inhibitory concentrations (MICs) were determined in minimal YNB medium containing ammonium sulfate (SA) as a nitrogen source, supplemented with 10 mM L-methionine (L-Met) when indicated, and RPMI-1640 medium, as recommended by CLSI in the M27-A3 procedure. The results are summarized in Table 2.

The D-penicillamine antifungal effect against *C. albicans* was previously studied in combination with fluconazole [33]. The authors discovered that D-Pen had synergistic effects with FLC, against not only planktonic cells, but also the biofilms of both sensitive and resistant *C. albicans* strains. The combined treatment increased the survival rate of *G. mellonella* larvae infected with *C. albicans*, as well as decreasing the fungal burden. Mechanism studies elucidated that the synergism is related to inhibition of the morphological transformation, the disruption of intracellular calcium homeostasis, and the activation of metacaspase, which is closely related to cell apoptosis. Our results also indicate that *Ca*Met2p inhibition could be related to that synergistic effect. 

We performed additional studies to test the susceptibility of *C. glabrata* clinical isolates to L-Pen. Six out of 8 examined strains were characterized as being resistant to fluconazole. All of the tested *C. glabrata* clinical isolates displayed similar susceptibilities to L-Pen as the wild-type strain in the YNB-SA medium (Table 3).

Unlike animals, fungal microorganisms are able to incorporate inorganic sulfur using an assimilatory mechanism that leads to L-cysteine biosynthesis in two different ways [34,35] (Figure 1). The major route for L-cysteine biosynthesis is the *O*-acetyl-L-serine pathway (OAS), which involves the condensation of sulfide with *O*-acetyl-L-serine. The alternative direct sulfhydrylation pathway condenses sulfide with *O*-acetyl-L-homoserine, yielding L-homocysteine, which is then converted to L-cystathionine and next to L-cysteine via the reverse transulfuration pathway. Filamentous fungi such as *Aspergillus nidulans* and *Neurospora crassa*, as well as many yeasts such as *C. albicans*, can synthesize L-cysteine using both ways, whereas *S. cerevisiae* and *C. glabrata* lack the *O*-acetyl-L-serine pathway [35,36]. This can be explained by the fact that *C. glabrata* is more closely related to *S. cerevisiae* than to *C. albicans*, as indicated from the phylogenetic analysis (Figure S6) performed using the BLAST program [37,38]. 

**Figure 8:**
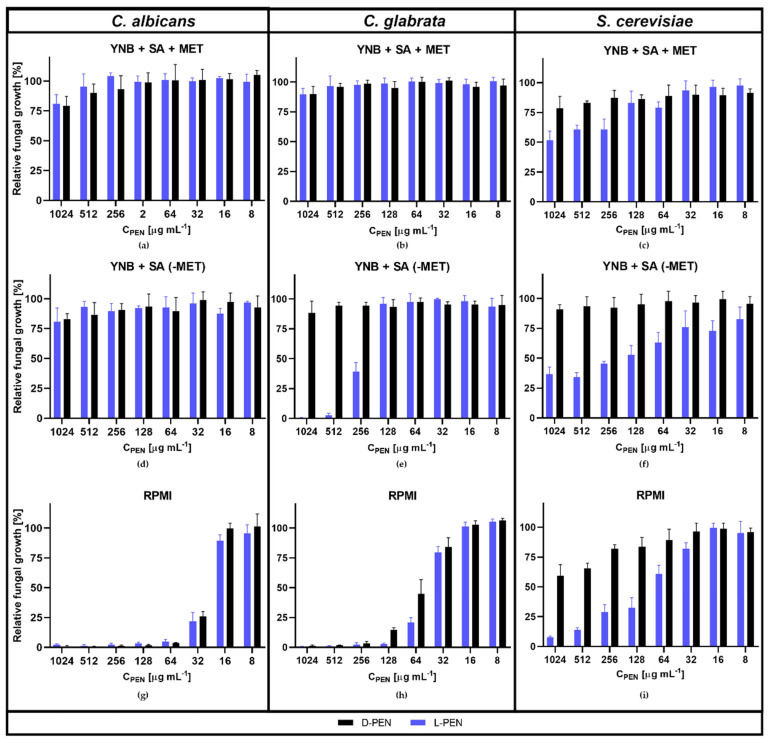
Inhibition of *C. albicans, C. glabrata*, and *S. cerevisiae* growth by L-Pen and D-Pen in YNB + SA ± 10 mM L-Met medium (**a**–**f**), and in RPMI medium (**g**–**i**). The error bars represent the standard deviation (SD).

Our results showed that L-Pen influenced the growth of *C. glabrata* and *S. cerevisiae* cells in minimal YNB medium (Figure 8). The growth of *C. glabrata* was inhibited by 50% via the addition of L-Pen at a concentration of 256 µg·mL^−1^ in a medium not supplemented with L-Met. This suggests that *C. glabrata* lacks a functional L-cysteine biosynthesis pathway from *O*-acetyl-L-serine, so that this amino acid can be only synthetized via the reverse transulfuration pathway from L-methionine (Figure 1). *S. cerevisiae* is another organism without the ability to produce L-cysteine from *O*-acetyl-L-serine, and in its case, our results showed that L-Pen did not cause complete growth inhibition in YNB; however, it did influence cell growth in 50% at 128 µg·mL^−1^, and the effect was dependent on the presence of L-Met. The addition of this amino acid reduced yeast cell sensitivity to the action of L-Pen (Figure 8). Moreover, the penicillamine concentrations determined as MIC_50s_ are comparable with those measured for *Ca*Met2p inhibition (256 and 128 µg·mL^−1^ correspond to the concentrations of ~1.72 mM and ~0.86 mM, respectively).

Interestingly, *C. albicans* cells possessing both the pathways mentioned above [39], are sensitive to L-Pen in RPMI-1640 medium, which mimics the human plasma physiological conditions with respect to amino acid content. The increased antifungal activity of L-Pen against the two other selected strains (Table 2) indicates the important role of amino acid permeases in the transport of L-Pen into the cell. Those permeases are extremely active in amino acid-rich media such as RPMI-1640.

In contrast to L-Pen, its D stereoisomer exhibited no antifungal activity in the YNB medium, regardless of L-Met supplementation (Table 2). However, similarly to L-Pen, D-Pen is active in RPMI-1640 medium (Table 2 and Figure 8g–i). This confirms the likely role of amino acid permeases in transporting both L- and D-Pen into the fungal cells. The general amino acid permease Gpa1p, present in fungi cells, is involved in the uptake of all of the proteinogenic L-amino-acids L-ornithine and L-citrulline, and some D-amino acids and toxic amino acid analogs [40]. It is also well-known that the expression of nitrogen permease-encoding genes such as GAP1 and the MEP genes are negatively influenced by the availability of favored nitrogen sources such as glutamine or ammonium sulfate [41]. The only available nitrogen source in YNB medium is ammonium sulfate. Therefore, the efficiency of L-Pen and D-Pen transport into cells is likely to be reduced in that medium, resulting in diminished antifungal activity. On the other hand, in amino acid-rich media like RPMI-1640, amino acid assimilation is stimulated [42].

**Figure 9:**
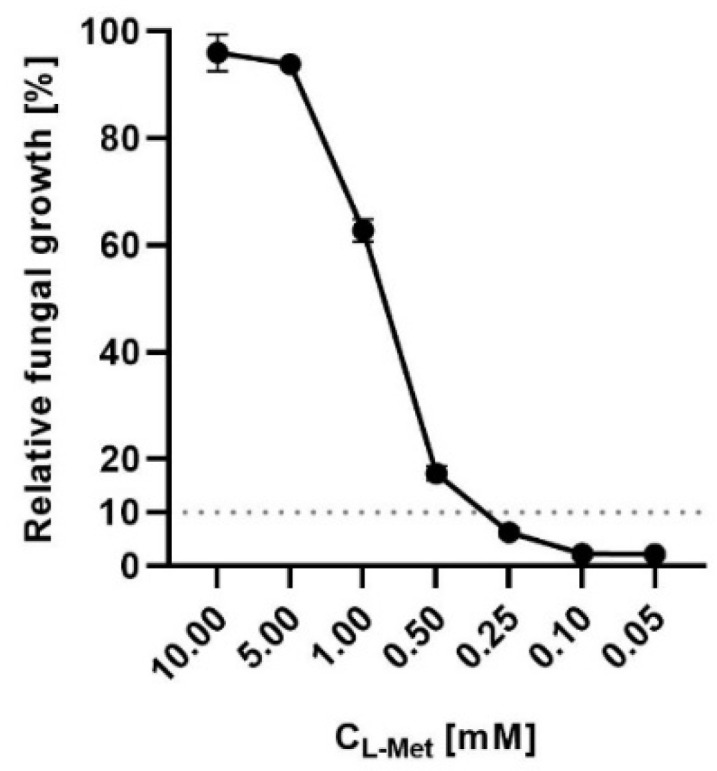
Inhibition of *C. glabrata* growth by L-Pen in YNB + SA medium supplemented with L-Met (10–0.05 mM). The dotted line represents 10% cell growth, which is a borderline reduction in visible growth, considered as no growth. The error bars represent the standard deviation (SD).

We have also examined L-Met concentration, which rescues the growth defect of *C. glabrata* cells treated with L-Pen in YNB medium supplemented with ammonium sulfate (Figure 9). 

The results showed that cell growth can be restored by the addition of approximately 0.3 mM L-Met to the medium, which is 10-fold more than the physiological L-Met concentration in the serum of healthy humans [43]. L-Met concentrations in a healthy individual’s serum tend to be stable, at a level of ~0.03 mM, independently on a habitual diet group [44]; however, abnormally increased L-Met serum concentrations reaching 0.6 mM can be developed in patients with liver diseases such as hepatic encephalopathy [45]. Our results thus indicate that targeting the Met2p enzyme from the L-Met biosynthetic pathway may lead to an antifungal effect in vivo. 

## 3. Materials and Methods

### 3.1. Reagents

All reagents were commercially available and purchased from Sigma-Aldrich, St. Louis, MO, USA. Reagents used for the determination of enzyme activity: AcCoA lithium salt, L-homoserine, guanidinium-HCl, and 5,5′-dithiobis(2-nitrobenzoic acid) (DTNB). Tested enzyme inhibitors included: acetyl-L-carnitine, D-penicillamine, L-penicillamine, 3-(2-thienyl)-L-alanine, L-methionine sulfoximine, DL-phosphinothricin, L-2,4-diaminobutyric acid dihydrochloride, DL-2-allylglycine, L-canavanine, *S*-(2-aminoethyl)-L-cysteine hydrochloride, and L-2-aminoadipic acid.

### 3.2. Microbial Strains and Growth Conditions

*Escherichia coli* One Shot^TM^ TOP10 cells (Invitrogen, Waltham, MA, USA) were used in the cloning procedures. *Escherichia coli* One Shot^TM^ BL21 Star^TM^ (*DE3*) and *Escherichia coli* Rosetta (*DE3*) *pLysS* competent cells were used to produce wild-type and recombinant proteins, respectively.

Antimicrobial activity of penicillamine was tested on 8 reference strains: *Candida albicans* ATCC 10231, *Candida parapsilosis* ATCC 22019, *Candida krusei* ATCC 6258, *Candida glabrata* ATCC 90030, *Candida famata* DSM 3428, *Candida rugosa* DSM 2031, *Candida dublinensis* CBS 7987, and *Saccharomyces cerevisiae* ATCC 9763; and 8 *C. glabrata* clinical isolates: *CZD 6*, CZD 209, GD 211, GD 310, CZD 342, CZD 373, CZD 377, and CZD 513. Clinical strains were isolated from patients from the Children’s Memorial Health Institute in Warsaw (CZD), or the Medical University of Gdansk (GD), in 2011 and 2012 [46]. 

All of the bacterial strains used were cultured on solid (with 1.5% (m/V) agar) and in liquid Luria-Bertani (LB) media (0.5% yeast extract, 1.0% peptone, 1.0% NaCl, supplemented with ampicillin (100 µg·mL^−1^) or chloramphenicol (34 µg·mL^−1^) when required), and incubated at 37 °C for 6–24 h.

Yeast strains were cultured on solid and in liquid YPG medium (1% yeast extract, 1% peptone, and 2% glucose; 1.5% agar in the case of solid medium) and grown at 30 °C for 18 h.

### 3.3. Cloning of MET2 Gene and Plasmids Construction

The *MET2* gene was amplified via polymerase chain reaction (PCR) using the *Candida albicans* SC5314 genome isolated with Genomic Mini AX YEAST kit (A&A biotechnology, Gdansk, Poland) as a matrix, and Phusion High-Fidelity PCR Master Mix with HF Buffer (Thermo Fisher Scientific, Waltham, MA, USA). Primers used for the amplification of the wild-type Met2p gene were 5′-CACCATGACATACAAAGACGTGACA-3′ as a forward primer and 5′-GATTATTCAATTATTCAAAAAACTAGTGATGAAAC-3′ as a reverse primer. Primers used for the amplification of the oligoHis-tagged Met2NHp gene were 5′-CACCATG**CATCATCATCATCATCAT**ACATACAAAGACG-3′ as a forward and 5′-TTGCATCCCTTGATTATTCAATTATTCAAAAAACTAGTGATG-3′ as a reverse primer; primers used for amplification of the oligoHis-tagged Met2CHp gene were 5′-CACCATGACATACAAAGACGTGACA-3′ as a forward and 5′-TCA**ATGATGATGATGATGATG**ATTATTCAAAAAACTAGT-3′ as a reverse primer; introduced poly-Histidine domains are marked in bold. The PCR temperature gradient profile was: initial denaturation at 98 °C for 10 s, 30 cycles of denaturation at 98 °C for 1 s, starter annealing at 58 °C for *MET2* and 68 °C for *MET2NH* for 5 s, and elongation at 72 °C for 30 s, followed by elongation at 72 °C for 60 s. The formed PCR products (1230 bp and 1248 bp for *MET2* and *MET2NH*, respectively) were cloned into the pET101/D-TOPO vector using the Champion™ pET101 Directional TOPO™ Expression Kit (Invitrogen), and transformed into *E. coli* One Shot^TM^ TOP10 cells (Invitrogen, Waltham, MA, USA). Plasmids were isolated with the use of the Plasmid Mini AX kit (A&A Biotechnology, Gdansk, Poland), and subjected to mutagenesis procedure. The constructed plasmids were sequenced in the coding region and transformed into *E*. *coli* One Shot^TM^ BL21 Star^TM^ (*DE3*) and *E. coli* Rosetta (*DE3*) *pLysS* competent expression cells in the case of the wild-type and His-tagged protein, respectively. 

### 3.4. Mutagenesis

The mutagenesis reaction mixture contained Phusion High-Fidelity PCR Master Mix with HF Buffer (Thermo Fisher Scientific, Waltham, MA, USA), and forward 5′P-CATTCCCCCCATGGATCCTCCA-3′ and reverse 5′P-GCATTAGAATACTCGGCAATTTACAACAATA-3′ primers, and pET101/D-TOPO +*MET2* or pET101/D-TOPO +*MET2NH* DNA matrix. The reaction mixture was subjected to PCR with a temperature gradient profile of an initial denaturation at 98 °C for 10 s, 30 cycles of denaturation at 98 °C for 1 s, starter annealing at 58 °C for 5 s, and elongation at 72 °C for 30 s, followed by elongation at 72 °C for 60 s. The obtained PCR products were digested with Fast Digest *Dpn*I restriction enzyme (Thermo Fisher Scientific, Waltham, MA, USA) in 37 °C for 30 min, and subjected to gel electrophoresis, after which undigested (7000 bp) fragments were cut out from the agarose gel and the DNA was isolated according to the Gen Elute™ Gel Extraction Kit protocol (Sigma-Aldrich, St. Louis, MO, USA). Isolated DNA was suspended in 50 µL water and used for a ligation procedure using T4 DNA Ligase Buffer (10×) (Thermo Fisher Scientific, Waltham, MA, USA) and T4 DNA Ligase (5 U·µL^−1^) (Thermo Fisher Scientific, Waltham, MA, USA). The ligation reaction was performed for 30 min in 22 °C, and the obtained plasmids containing mutated *MET2* and *MET2NH* genes were used for the transformation of *E. coli* One Shot^TM^ TOP10 (Invitrogen, Waltham, MA, USA) competent cells.

### 3.5. Western Blot Analysis

Proteins separated in 10% polyacrylamide gel were subjected to electroblotting to a nitrocellulose membrane for 1 h at 20 mA. A nitrocellulose membrane with transferred proteins was then placed in 5% skim milk wash buffer solution (10 mM Tris-HCl, pH 8.0, 30 mM NaCl) and left overnight. The membrane was washed 3 times with 10% (by volume) wash buffer, and next incubated for 1 h in antibody solution (10 mM Tris-HCl, pH 8.0, 30 mM NaCl, 1% (m/V) BSA, 0.05% (by volume) Tween20, anti-His6 0.033% (by volume) antibodies (Monoclonal Anti-polyhistidine-Peroxidase antibody A7 058-1VL (Sigma-Aldrich, St. Louis, MO, USA))). The detection of proteins conjugated to antibodies was made with 1 mL of 3,3′,5,5′-tetramethylbenzidine (TMB) liquid substrate system for membranes (Sigma-Aldrich, St. Louis, MO, USA).

### 3.6. Expression of MET2 Gene

The constructed plasmids pET101/D-TOPO + *MET2* and pET101/D-TOPO + *MET2NH* were transformed into *E. coli* One Shot^TM^ BL21 Star^TM^ (*DE3*) and *E. coli* Rosetta (*DE3*) *pLysS* competent cells, respectively. Expression cells were grown overnight at 37 °C in LB medium supplemented with ampicillin and chloramphenicol in the case of *E. coli* Rosetta (*DE3*) *pLysS* cells. A total of 10 mL of starter culture was added to 800 mL of LB medium supplemented with ampicillin and chloramphenicol, and incubated at 37 °C up to an optical density (OD_600_) = 1.0. Induction was made with isopropyl-β-d-thiogalactoside (IPTG) to a final concentration of 1 mM. Cells were cultured for 6 h at 37 °C. Cells were harvested via centrifugation for 20 min at 4000 rpm in 4 °C. The obtained cell pellets were stored for later use at −20 °C.

### 3.7. Enzyme Purification

The bacterial pellet was suspended in buffer 1 (20 mM sodium phosphate buffer pH 7.0) supplemented with cOmplete™ Protease Inhibitor Cocktail (Hoffmann-La Roche, Basel, Switzerland), 10 mM EDTA, and 10 mM dithiothreitol (DTT). The mixture was sonicated on ice (Branson digital sonifier W-250 D, Danbury, CT, USA) and centrifuged for 20 min at 10,000 rpm. Streptomycin sulphate was added the supernatant to a final concentration of 1.2%, and the mixture was centrifuged for 20 min at 12,000 rpm at 4 °C. The obtained supernatant was saturated with 36.2% ammonium sulphate, mixed in an ice bath for 20 min, and then centrifuged for 20 min at 12,000 rpm at 4 °C. During this step, the protein mixture was initially purified, and the precipitate containing the examined proteins was dissolved in buffer 1 containing 40 mM NaCl and 10 mM MgCl_2_, and centrifuged for 20 min at 12,000 rpm in 4 °C. To dispose of the excess salt in the mixture, 20% polyethylene glycol 6000 (Sigma-Aldrich, St. Louis, MO, USA) was added and centrifuged for 20 min at 12,000 rpm in 4 °C. The cell pellet was suspended in 10 mL buffer 1 supplemented with cOmplete™ Protease Inhibitor Cocktail (Hoffmann-La Roche, Basel, Switzerland), 10 mM EDTA, and 10 mM DTT. The solution was centrifuged and loaded onto the Resource™ Q column (GE Healthcare, Chicago, IL, USA). The elution of the proteins was conducted in a rising concentration of NaCl up to 0.5 M concentration in elution buffer 2 (20 mM phosphate buffer of pH 7.0 and 1 M NaCl) in 10 column volumes.

The bacterial pellet was suspended in binding buffer W_5_ (20 mM sodium phosphate buffer pH 8.0, 5 mM imidazole, 500 mM NaCl, and 1 mM Tween 20) supplemented with cOmplete™ Protease Inhibitor Cocktail (Hoffmann-La Roche, Basel, Switzerland) and 10 mM DTT. The mixture was sonicated on ice (Branson digital sonifier W-250 D, Danbury, CT, USA) and centrifuged for 20 min at 10,000 rpm. A total of 10 mL of supernatant was loaded onto a 5 mL HisTrap™ Fast Flow (GE Healthcare, Chicago, IL, USA) column equilibrated with binding buffer. Elution was performed in a linear rising imidazole concentration. Elution buffer W_500_: 20 mM sodium phosphate buffer pH 8.0, 500 mM imidazole, 500 mM NaCl, and 1 mM Tween 20 (elution program: 0–6 min, 0% B, 6–30 min, 0–50% B, linear gradient, flow rate 3 mL·min^−1^, 0.3 MPa, detector UV (l = 254, 280 nm)). 

Densitometric analysis measuring the purity rate of the purified protein was performed with the Gel analyzer program [47].

### 3.8. Determination of Met2p Activity

The assay was conducted according to the method of Foyn et al. [48]. A total of 300 nM of isolated enzyme supplemented with 20% (by volume) of glycerol was used in the assay. Standard incubation mixtures contained 3.5 mM AcCoA lithium salt and 10 mM L-homoserine in 50 mM sodium phosphate buffer (pH 8.0) containing 100 mM NaCl and 1 mM EDTA in a total volume of x mL. The reaction was commenced via the addition of the enzyme (x μL of enzyme solution, 300 nM in 20% glycerol) and performed for 5 min at 37 °C. The reaction was stopped via the addition of 100 µL of quenching buffer (100 mM sodium phosphate buffer (pH 5.8), 3.2 mM guanidinium-HCl). The amount of CoA formed was quantified via the addition of 20 µL of 3 mM 5,5′-dithiobis(2-nitrobenzoic acid) (DTNB) dissolved in 100 mM sodium phosphate buffer (pH 6.8) containing 10 mM EDTA. A total of 150 µL of the mixture was transferred to a 96-well plate and measured spectrophotometrically at 412 nm with a microplate reader (TECAN Spark 10M, Grödig, Austria).

### 3.9. Determination of the Kinetic Parameters

The kinetic parameters were determined for both the wild-type and the His-tagged version of the enzyme by measuring the activity of 300 nM protein according to the activity assay procedure. The reaction mixture contained various concentrations of substates: 0–4 mM AcCoA and 0–6 mM L-Hom at a constant concentration of other substrates, 10 mM L-Hom and 3.5 mM AcCoA, respectively. 

### 3.10. Determination of the Optimal pH and Reaction Buffer

The optimal pH for the enzyme activity was determined according to the activity assay procedure with the changing pH of the buffers used: Tris-HCl (pH 8.5–9.0), HEPES (pH 7.0–8.0), NaHPO_4_ (pH 6.0–8.0), and glycine (pH 8.5–11.0). The determination of the optimal reaction buffer was made by comparing the activity of the enzyme in the above reaction buffers at pH 8.0–8.5. 

### 3.11. Inhibitory Assay

For the assessment of the inhibitory assay, 1–15 mM of inhibitor was added to the activity reaction buffer. The reaction was commenced via the addition of 300 nM of *Ca*Met2NHp. The influence of the inhibitor on the activity of the enzyme was determined by the measurement of the difference in the absorbance at 412 nm, assessed according to the above method. 

### 3.12. Determination of Minimum Inhibitory Concentration

Minimum inhibitory concentrations (MICs) were performed using the modified M27-A3 procedure specified by the CLSI [49]. In 96-well plates, solutions of L-penicillamine (Sigma-Aldrich, St. Louis, MO, USA) and fluconazole (Sigma-Aldrich, St. Louis, MO, USA) were serially diluted and inoculated with overnight-tested fungal strains to a final concentration of ~10^4^ colony-forming units (CFU)/mL in a Yeast Nitrogen Base (YNB) without amino acids and ammonium sulfate (Sigma-Aldrich, St. Louis, MO, USA), supplemented with or without 0.05–10 mM L-methionine and Roswell Park Memorial Institute medium 1640 (RPMI) (Sigma-Aldrich, St. Louis, MO, USA). Plates were incubated at 37 °C for 24 h; the rate of growth was determined by measuring the optical density at 600 nm using a microplate reader (TECAN Spark 10M, Grödig, Austria). The MIC_50_ and MIC_90_ parameters were defined as the lowest concentration of antifungal compound that inhibited fungal growth by at least 50% or 90%, respectively. 

### 3.13. Homology Modeling and Docking Analysis

The structure of the enzyme from *M. smegmatis* (PDBID: 6IOH) was used as a template to prepare the AlphaFold model of the *C. albicans* enzyme [20]. The crystal structure of the same enzyme (PDBID: 6IOH), as well as the structure of the AlphaFold model of the *C. albicans* enzyme were used as the receptors for the docking analysis. The structures of all ligands were built using the HyperChem software [50]. All docking calculations were then performed via the Autodock 4.2 package, with a modified forcefield [51,52] after preparing all of the parameter files and processing the ligands, as well as the receptor structures, through the Autodock utility scripts. To avoid any bias toward the correct structure of the known X-ray complex, the ligand used for redocking and protocol validation (L-HOM) found in the pdb file was not used directly, but it was rebuilt from scratch using the same protocol as was used for building all of the other ligands. Since the preliminary calculations showed that water molecules present in the binding site might be important for interactions with the ligand, the water molecules that were within 3.3 Å radius and had the lowest B-factor values were considered as part of the binding site.

The center of the docking grid was set to the geometric center of the bound ligand and its size was set to 40 Å in each direction, to cover not only the binding site, but also the majority of the protein subunit. The adequacy of the docking protocol, as well as the forcefield used, was verified by redocking of the rebuilt L-HOM, as mentioned earlier. The geometry of the native complex was restored, with the average RMS of the lowest energy cluster below 1.5 Å. Figures showing the results of molecular modeling were prepared using the VMD program [53].

## 4. Conclusions

In conclusion, the *MET2* gene of *C. albicans* was unequivocally identified as coding for L-homoserine *O*-acetyltransferase, catalyzing the first step in the L-Met biosynthesis pathway. The Ca*MET2* was cloned and expressed in *E. coli* as native and His-tagged fusion proteins. The recombinant *Ca*Met2p was only slightly inhibited by the end pathway product, i.e., L-Met, at a non-physiological concentration; thus, the mechanism of feedback inhibition seems not to play any role in amino acid biosynthetic pathway regulation. The L- and D-penicillamine inhibitory potential against *Ca*Met2NHp, as well as both compounds’ antifungal activities, prove the usefulness of Met2p as a molecular target for antifungal chemotherapy. For this reason, the development of inhibitors with a stronger activity than this displayed by L-Pen and D-Pen is worth trying.

## Figures and Tables

**Table 1:** Kinetic parameters of *C. albicans* Met2p and Met2NHp. Value of k_cat_ were calculated per monomer. Data shown are the means of at least three independent experiments ± SD.

Substrate	K_M_ [mM]	k_cat_ [s^−1^]	k_cat_/K_M_ [M^−1^·s^−1^]	V_max_ [µmol·min^−1^·mg^−1^]
	***Ca*Met2p**			
L-Hom	0.405 ± 0.050	13.0 ± 0.405	32.1 × 10^3^	17.1 ± 0.536
AcCoA	0.906 ± 0.119	15.2 ± 0.572	16.8 × 10^3^	20.1 ± 0.756
	***Ca*Met2NHp**			
L-Hom	0.578 ± 0.118	13.1 ± 0.723	22.7 × 10^3^	17.2 ± 0.956
AcCoA	1.13 ± 0.150	15.0 ± 0.635	13.3 × 10^3^	19.8 ± 0.839

**Table 2:** Susceptibility of fungal strains to L-Pen, D-Pen, fluconazole (Flu), and amphotericin B (AmB). MIC_50_ (MIC_90_) are the compound concentrations at which growth is inhibited by 50% or 90%, respectively. > Indicates no activity at the mentioned concentration. The experiments were performed in at least three replicates. * Data retrieved from [32].

	MIC_50_ (MIC_90_) [µg·mL^−1^]				
	L-Pen			Flu	AmB
Strain	YNB + SA + L-Met	YNB + SA − L-Met	RPMI1640	RPMI1640	RPMI1640
*Candida albicans* ATCC 10231	>1024	>1024	32 (64)	(4)	(0.5)
*Candida parapsilosis* ATCC 22019	>1024	>1024	256 (1024)	(8)	(1)
*Candida krusei* ATCC 6258	>1024	>1024	>1024	(64)	(1)
*Candida glabrata* ATCC 90030	>1024	256 (512)	64 (128)	(32)	(1)
*Candida famata* DSM 3428	>1024	>1024	1024 (>1024)	(16)	(1.0) *
*Candida rugosa* DSM 2031	>1024	>1024	1024 (>1024)	(16)	(2.0) *
*Candida dublinensis* CBS 7987	>1024	>1024	>1024	(4)	(0.25) *
*Saccharomyces cerevisiae* ATCC 9763	512 (>1024)	128 (>1024)	128 (1024)	(8)	(0.5)
	**D-Pen**			**Flu**	**AmB**
	**YNB + SA** **+ L-Met**	**YNB + SA − L-Met**	**RPMI1640**	**RPMI1640**	**RPMI1640**
*Candida albicans* ATCC 10231	>1024	>1024	32 (64)	(4)	(0.5)
*Candida glabrata* ATCC 90030	>1024	>1024	128 (256)	(32)	(1)
*Saccharomyces cerevisiae* ATCC 9763	>1024	>1024	>1024	(8)	(0.5)

**Table 3:** Susceptibility of *C. glabrata* clinical isolates to L-Pen and Flu. MIC_90_ (MIC_50_), minimal inhibitory concentrations at which 90% (50%) of cells growth was inhibited. > Indicates no activity at the concentration mentioned; R, resistant to fluconazole; S, sensitive to fluconazole. The experiments were performed with at least three replicates.

	Susceptibility	MIC_90_ (MIC_50_) [µg·mL^−1^]	
Strain	Flu	Flu	L-Pen
*C. glabrata* CZD 6	S	32	512 (256)
*C. glabrata* CZD 209	R	>256	512
*C. glabrata* GD 211	R	256	512 (256)
*C. glabrata* GD 310	R	>256	512
*C. glabrata* CZD 342	S	16	512
*C. glabrata* CZD 373	R	256	512 (256)
*C. glabrata* CZD 377	R	>256	512
*C. glabrata* CZD 513	R	>256	512

## Data Availability

Data supporting the reported results are presented in this manuscript and its supplemental material. Raw data supporting our results are also available as data sets: 1. Gabriel, I., Kuplińska, A., Rząd, K., and Milewski, S. (2022). Identification and cloning of *C. albicans* SC5314 genes encoding L-methionine biosynthetic pathway enzymes (data set). Gdańsk University of Technology. https://doi.org/10.34808/zac2-tz70 (accessed on 18 January 2022); 2. Gabriel, I., Kuplińska, A., Rząd, K., and Milewski, S. (2022). Overproduction of homoserine *O*-acetyltransferase (CaMet2p) native and His-tag versions (data set). Gdańsk University of Technology. https://doi.org/10.34808/kgrw-6e43 (accessed on 21 June 2022); 3. Gabriel, I., Kuplińska, A., Rząd, K., and Milewski, S. (2022). Antifungal activity of L-homoserine *O*-acetyltransferase (CaMet2p) inhibitors (data set). Gdańsk University of Technology. https://doi.org/10.34808/jhc2-ad98 (accessed on 5 July 2022).

## Supplementary Materials

The following supporting information can be downloaded at: https://www.mdpi.com/article/10.3390/ijms23147763/s1.
