# Supplementary material for: Antifungal Effect of Penicillamine Due to the Selective Targeting of L-Homoserine O-Acetyltransferase
Source: Int J Mol Sci. 2022 Jul 14;23(14):7763. doi: 10.3390/ijms23147763 (PMC9317633; doi:10.3390/ijms23147763)
Supplement: Supplementary file 1 [file ijms-23-07763-s001.zip › ijms-1789208-supplementary.pdf]

Article

Antifungal effect of Penicillamine due to the selective targeting of L-homoserine O-acetyltransferase

Aleksandra Kuplińska, Kamila Rząd, Marek Wojciechowski, Sławomir Milewski and Iwona Gabriel*

Department of Pharmaceutical Technology and Biochemistry, Gdansk University of Technology, Gdansk, Poland; aleksandra.kuplinska@pg.edu.pl (A.K.); kamila.rzad@pg.edu.pl (K.R.); marek.wojciechowski@pg.edu.pl (M.W.); slawomir.milewski@pg.edu.pl (S.M.); iwona.gabriel@pg.edu.pl (I.G.) * Correspondence: iwona.gabriel@pg.edu.pl (I.G.); Tel.: +48-58-348-6078 ; Fax: +48-58-347-1144

<u>**Table of contents:**</u>

1. **The multiple sequence alignment of amino acids sequences from 60 microorganisms from the *Ascomycota* group ……………..…………………………………………………….………2**
2. **Phylogenetic tree of amino acid sequences from 60 microorganisms from the *Ascomycota* group …………………………..………………………………………….…………….……7**
3. **Conservation of characteristic amino acid motifs in L-homoserine O-acetyltransferase sequences…………………………………………………………………….……………….8**
4. **Overproduction of *Ca*Met2p and *Ca*Met2NHp in *E. coli* cells…………………….…………8**
5. **SDS-PAGE or Western blot analysis of enzymes overproduction and purification……….....9**
6. **Phylogenetic tree of Met2p from various organisms……………………………………..…..9**
7. **References…………………………………………………………………….…….…..……9**

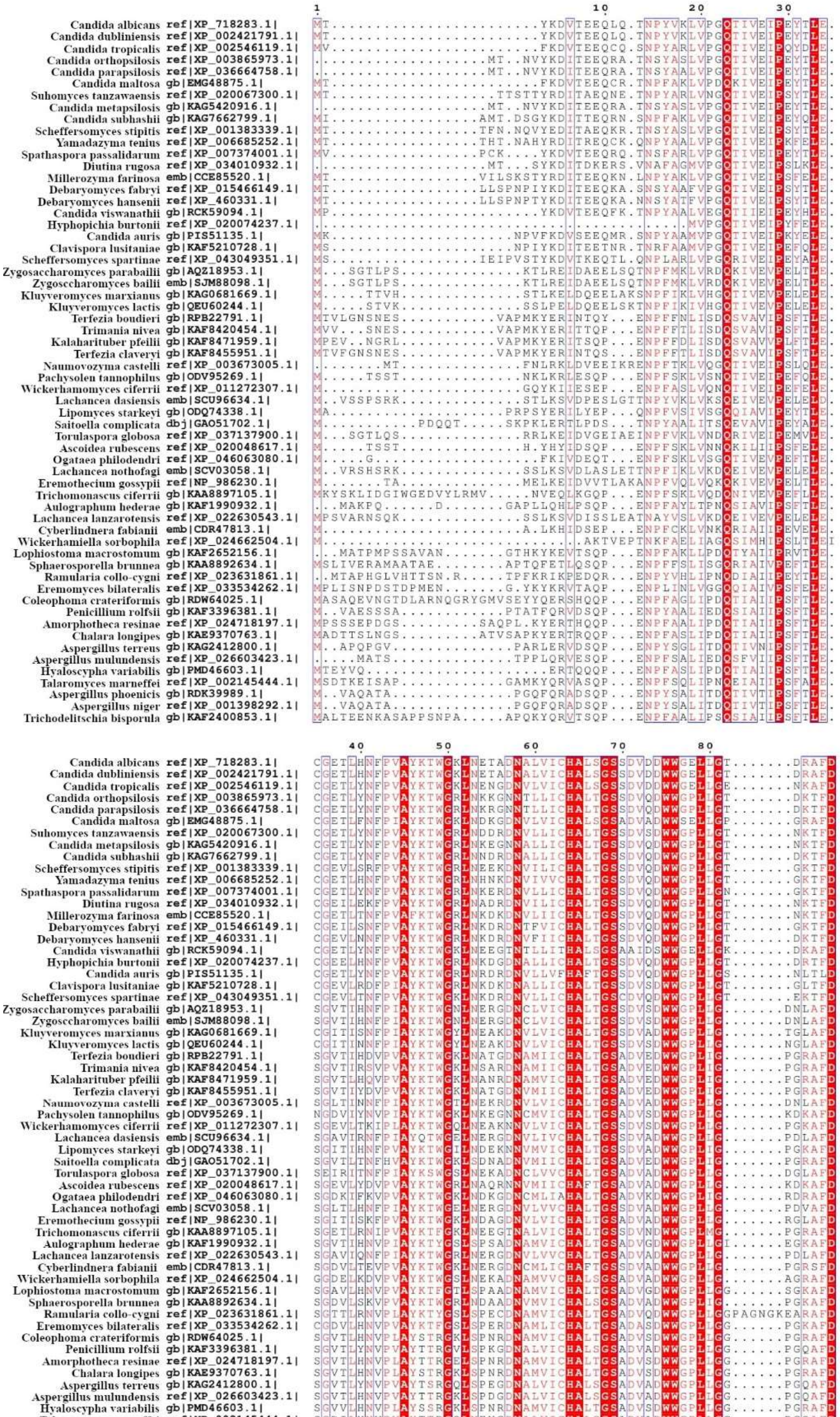

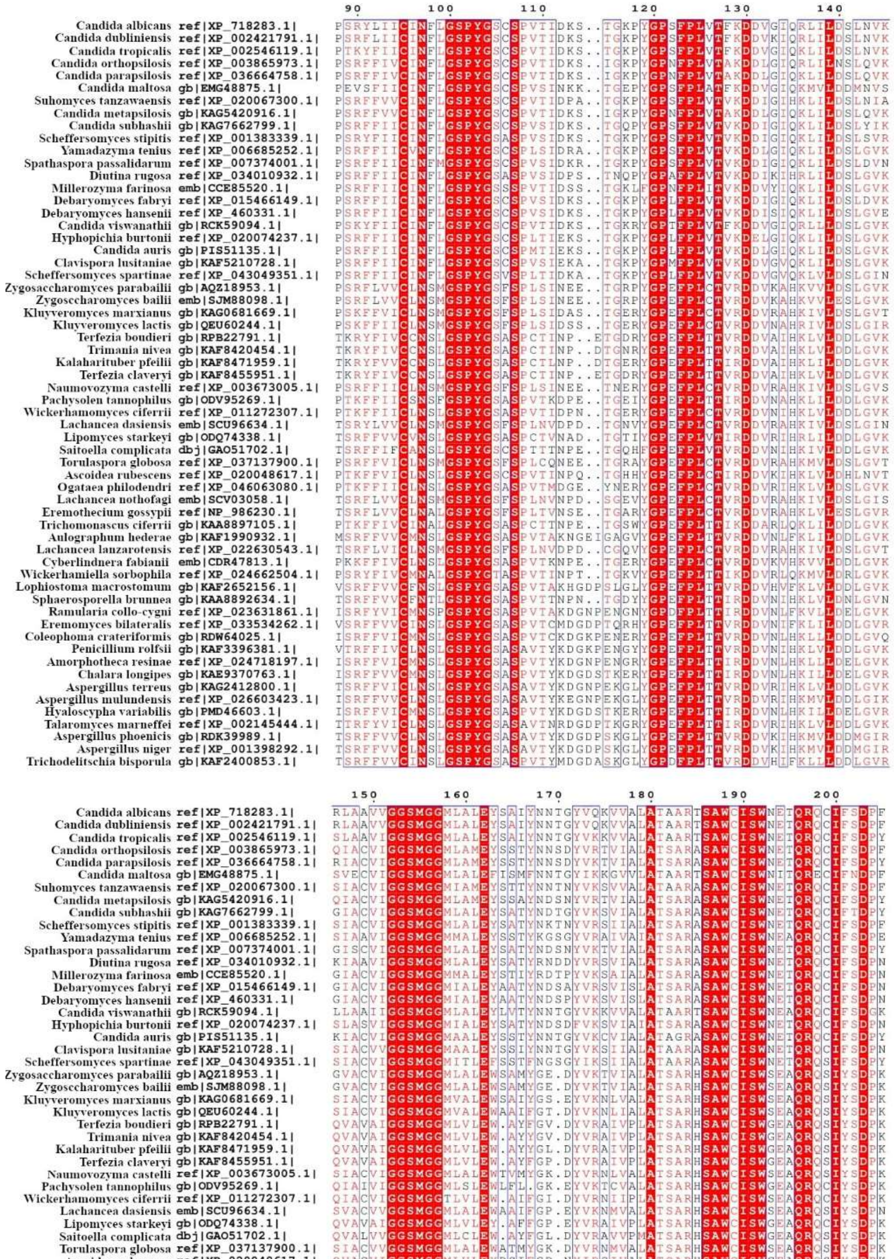

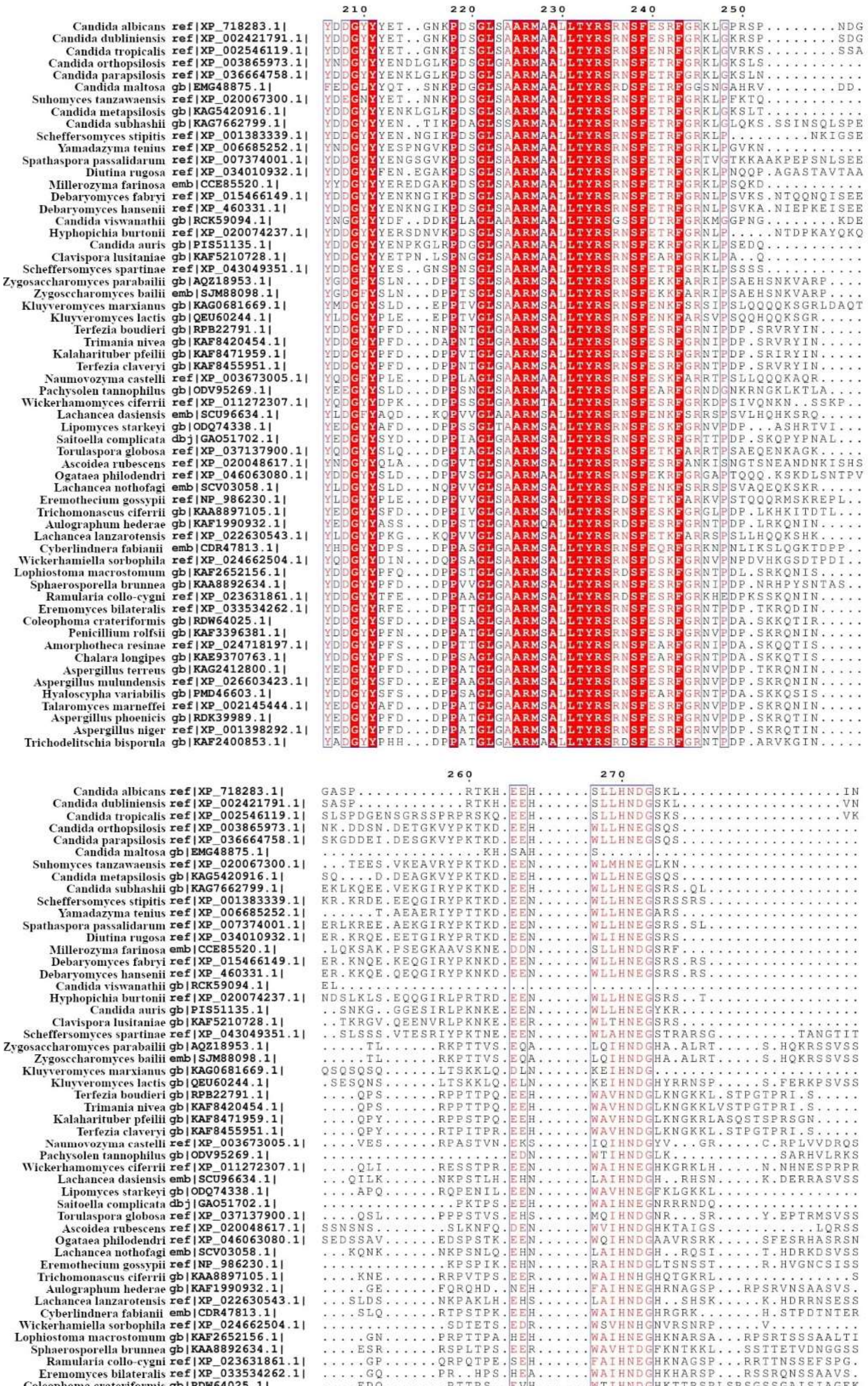

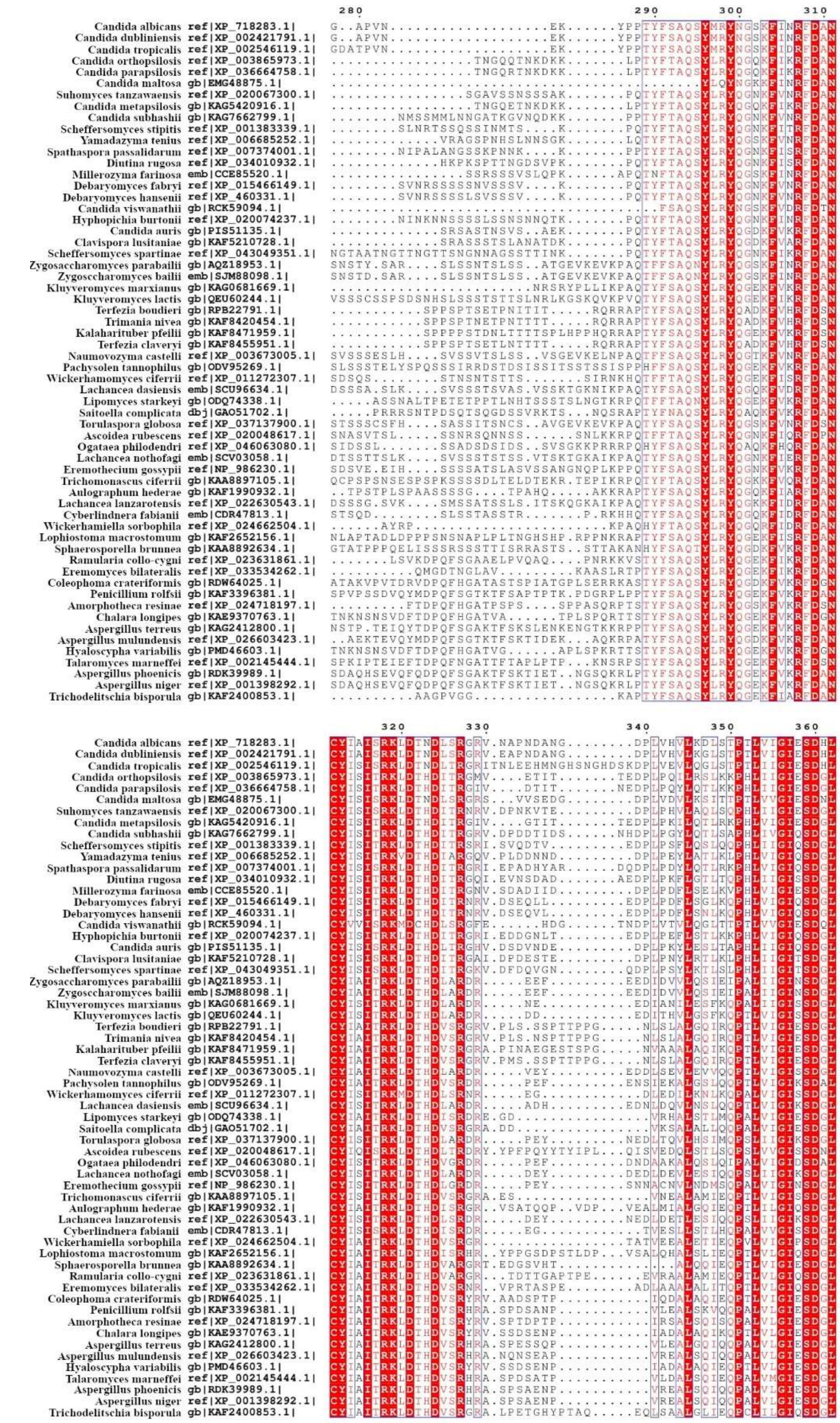

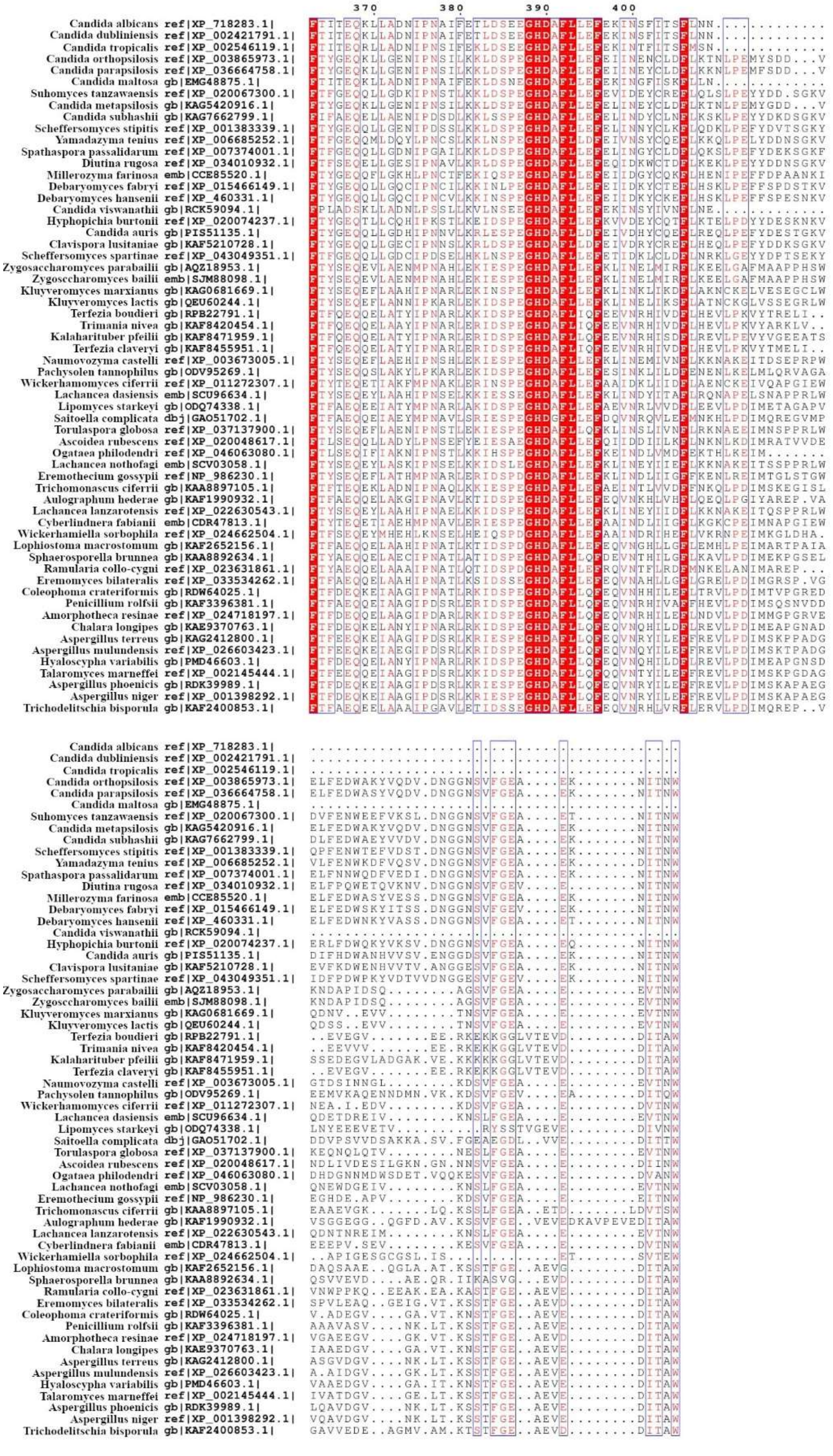

Figure S1. The multiple sequence alignment of amino acid sequences from 60 microorganisms from the *Ascomycota* group to the *Ca*Met2 amino acid sequence from *C. albicans*. Analysis performed using BLAST program [1].

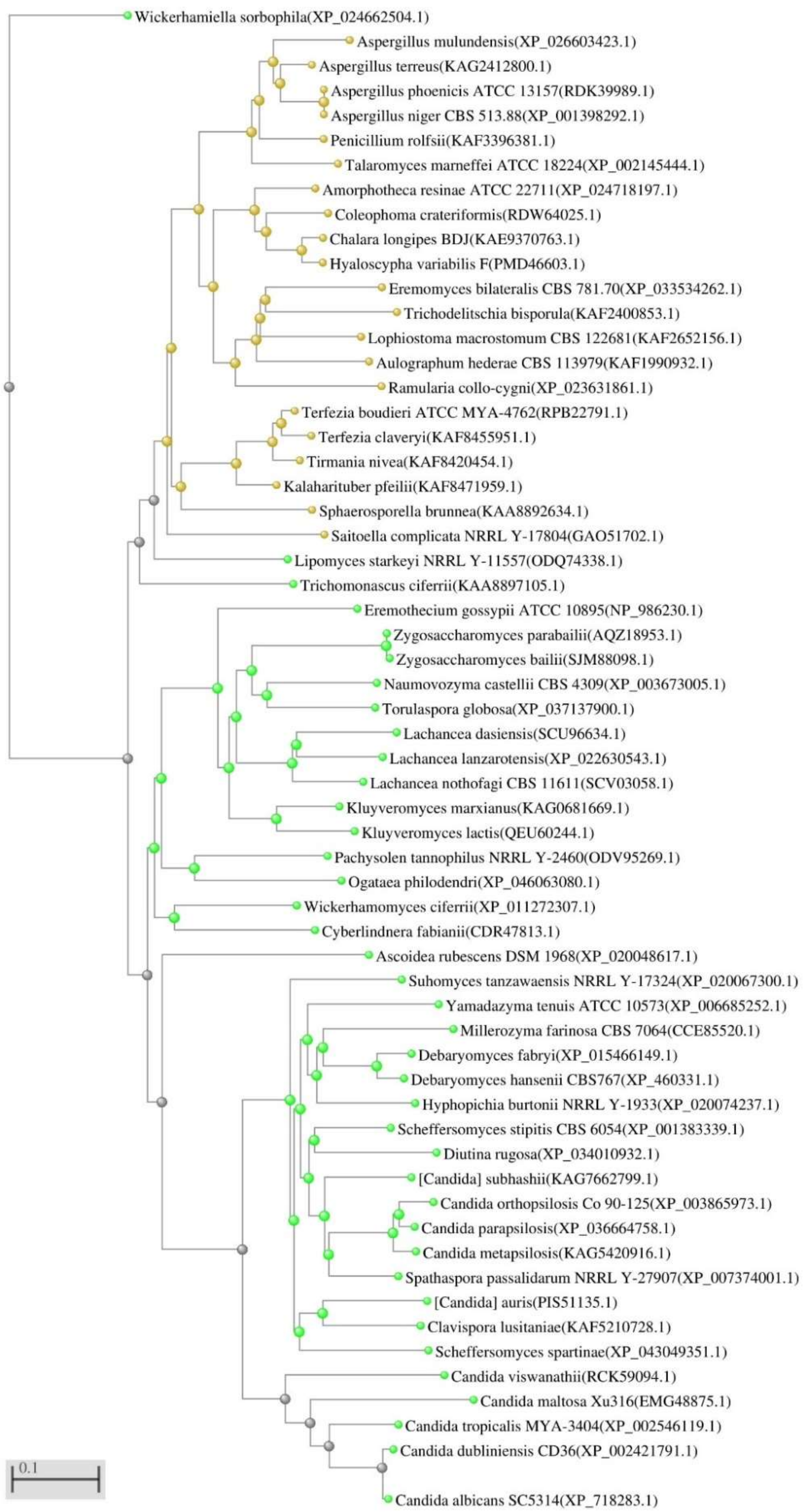

Figure S2. Phylogenetic tree of amino acid sequences from 60 microorganisms from the Ascomycota group with high similarity (≥50,34 % of identity) to the Met2 amino acid sequence from *C. albicans*. Analysis performed using BLAST program [1].

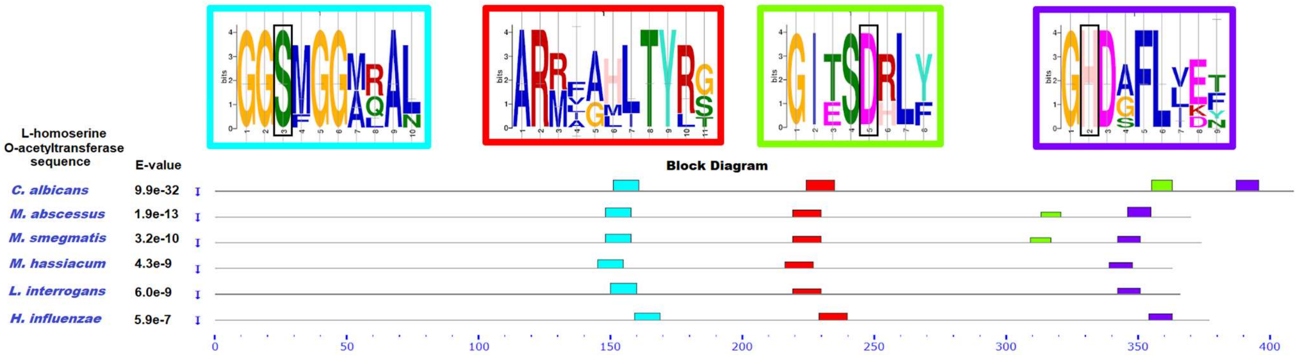

Figure S3. Conservation of characteristic amino acid motifs in L-homoserine O-acetyltransferase sequences. The catalytic triad residues are marked with a black frame. The analysis was performed using MAST , FIMO and TOMTOM tools from The MEME suite [2–4].

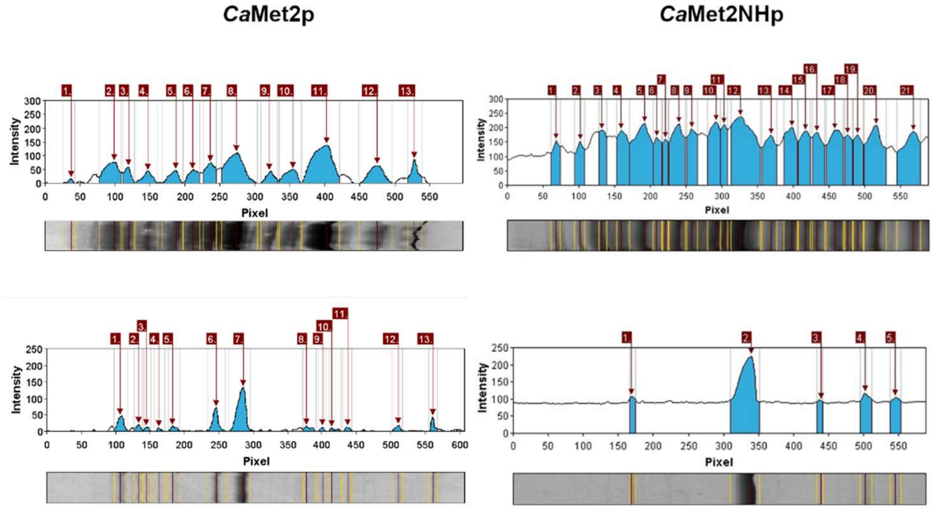

(a)

Protein name	*Ca*Met2p	*Ca*Met2NHp
Total amount of protein in cell extract	100 %	100%
Rate of protein expression	23 %	19 %
Volume of purified protein	10 mL	10 mL
Concentration of recombinant protein	0.114 mg mL^{-1}	0.237 mg mL^{-1}
Purity of recombinant protein	44 %	86 %

(b)

Protein name	*Ca*Met2p	*Ca*Met2NHp
Theoretical molecular mass	45.4 kDa	46.2 kDa
Calculated molecular mass after SDS-PAGE analysis	45.0 kDa	46.6 kDa

(c)

Figure S4. Overproduction of *Ca*Met2p and *Ca*Met2NHp in *E. coli* cells: **(a)** Densitometric analysis of SDS-PAGE electrophoresis analysis of cell extract (top) and purified fraction (bottom) from *E. coli* cells producing *Ca*Met2p and *Ca*Met2NHp recombinant protein. Gel Analyzer software [5]; **(b)** Mass balance of *Ca*Met2p and *Ca*Met2NHp purification developed on the base of the Gel Analyzer Program analysis results and experimental work; **(c)** Theoretical molecular mass (ProtParam analysis [6]) and determined by SDS-PAGE analysis molecular mass of the *Ca*Met2p and *Ca*Met2NHp.

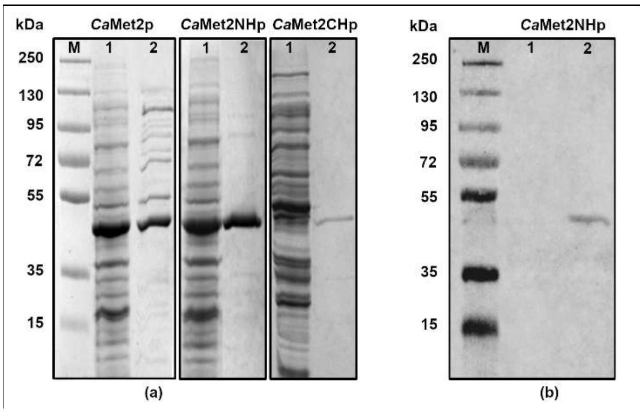

Figure S5. (a) SDS-PAGE analysis of CaMet2p, CaMet2NHp and CaMet2CHp overproduction in E. coli cells and purification. Plus Prestained Protein Ladder (M), cell-free extract (1), CaMet2p-containing fraction (2) (b) Western blot analysis of CaMet2NHp overproduction. (1) no IPTG induction, (2) IPTG induced.

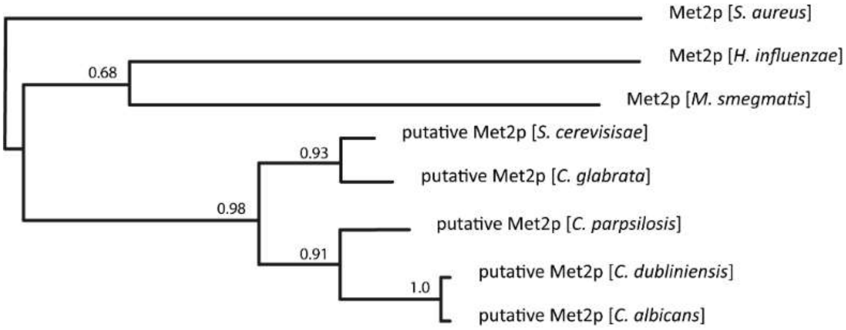

Figure S6. Phylogenetic tree of Met2p from various organisms. Analysis performed using BLAST program [1].

References

1. Altschul, S.F.; Gish, W.; Miller, W.; Myers, E.W.; Lipman, D.J. Basic local alignment search tool. *J. Mol. Biol.* **1990**, *215*, 403–410, doi:10.1016/S0022-2836(05)80360-2.
2. Bailey, T.L.; Gribskov, M. Combining evidence using p-values: Application to sequence homology searches. *Bioinformatics* **1998**, *14*, 48–54, doi:10.1093/bioinformatics/14.1.48.
3. Gupta, S.; Stamatoyannopoulos, J.A.; Bailey, T.L.; Noble, W. Quantifying similarity between motifs. *Genome Biol.* **2007**, *8*, R24, doi:10.1186/gb-2007-8-2-r24.
4. Grant, C.E.; Bailey, T.L.; Noble, W.S. FIMO: scanning for occurrences of a given motif. *Bioinformatics* **2011**, *27*, 1017–1018, doi:10.1093/bioinformatics/btr064.
5. Lazar Jr., I.; Lazar Sr., I. GelAnalyzer 19.1 (www.gelanalyzer.com).
6. Gasteiger, E.; Hoogland, C.; Gattiker, A.; Duvaud, S.; Wilkins, M.R.; Appel, R.D.; Bairoch, A. Protein Identification and Analysis Tools on the ExPASy Server. In *The Proteomics Protocols Handbook*; Humana Press, 2005; pp. 571–607.
